# Is There any Time Dependant Echocardiographical Finding in Chronic Hemodialysis Patients?

**DOI:** 10.4021/cr241e

**Published:** 2012-11-20

**Authors:** Mohsen Abbasnezhad, Hamid Tayyebi-Khosroshahi, Amin Ghanbarpour, Afshin Habibzadeh

**Affiliations:** a Dept. of Cardiology, Cardiovascular Research Center, Tabriz University of Medical Sciences, Tabriz, Iran; b Dept. of Nephrology, Tabriz University of Medical Sciences, Tabriz, Iran; c Medical Philosophy and History Research Center, Tabriz University of Medical Sciences, Tabriz, Iran

**Keywords:** Hemodialysis, Echocardiography, Left ventricle hypertrophy

## Abstract

**Background:**

Cardiac disease is the main cause of death in hemodialysis patients. In hemodialysis patients cardiovascular complications are great clinical challenge, and function, shape and left ventricle abnormalities are present in 70 - 80 percent of dialysis patients. Changes in heart function occur in hemodialysis period and are effective in patient’s prognosis. In this study we aim to evaluate time dependant clinical and echocardiographical findings in chronic hemodialysis patients.

**Methods:**

In a cross-sectional study, 100 adult hemodialysis patients (51% male and 49% female with mean age 52.13 ± 12.69 years) visiting dialysis unit in Imam Reza and Madani hospitals between years 2010 and 2011 were studied in group 1 (hemodialysis ≤ 6 months), group 2 (hemodialysis for 6 months to 3 years) and group 3 (hemodialysis ≥ 3 years). Demographic, laboratory and echocardiographic findings were compared between groups.

**Results:**

Among demographic findings, group 3 had significantly higher diastolic blood pressure and weight gain and was older than other two groups (P < 0.05). By increase in hemodialysis period, patients had higher blood urea nitrogen and lower serum albumin levels (P < 0.05). Potassium level in group 2 was significantly higher than group 3 and that was higher than group 1. There was no difference between groups in left ventricular hypertrophy (LVH), left atrium dilatation, ejection fraction and mitral insufficiency. Diastolic dysfunction increased in line with increase in hemodialysis period (P = 0.007). Hemodialysis period was higher in patients with LVH than those without (34.80 ± 9.2 months versus 18.51 ± 2.22 months, P = 0.01).

**Conclusion:**

In hemodialysis patients, diastolic dysfunction increases by the hemodialysis time (years). LVH and LA dilation also increase during time, but not significantly.

## Introduction

Cardiovascular complications are the most important cause of death in patients with end stage renal disease (ESRD) on hemodialysis treatment [1, 2]. These patients have an increased prevalence of coronary heart disease, silent myocardial ischemia, ventricular and supraventricular disturbances in heart rhythm [3], left ventricular hypertrophy, changes in the mitral and aortic valves, and enlargement of the left atrium [4, 5].

Anomalies of left ventricular (LV) structure and function are very frequent among CKD patients [6]. Many data are available about patients with advanced renal dysfunction. Among patients with ESRD, nearly 15% have systolic dysfunction, nearly 40% have heart failure and more than 70% have LVH [7, 8].

The diagnosis of LV abnormalities by Doppler echocardiography is an important step for the characterization of individuals with higher cardiovascular risk, estimating the prevalence of primary heart disease in a population to study its predisposing factors, prognostic impact and the effect of therapeutic interventions [9]. Echocardiography provides a non-invasive assessment of left ventricular structure and function. Echocardiography also provides information on both left ventricular geometry and left ventricular contractility. The type of left ventricular geometry may also have prognostic implications [10]. It has been shown that regression of electrocardiographic LV hypertrophy improves prognosis in the general population [11]; as well regression of LV abnormalities is associated with improved cardiac outcome in dialysis patients [12].

Cardiac function changes in time in hemodialysis patients and diagnosing these changes could be effective in prognosis of the disease. In this study we evaluate the time dependant clinical and echocardiographical findings in chronic hemodialysis patients.

## Methods

In this cross-sectional study 100 adult hemodialysis patients referring to hemodialysis ward in Imam Reza hospital and Shahid Madani hospital, Tabriz, Iran were studied. All adult patients with no known valvular heart disease and no history of previous coronary intervention were included. Informed consent was obtained from all patients, and the study was carried out following the principles of the Helsinki Declaration (Edinburgh Amendment, 2000).

Patients divided into three groups according to the time they have started hemodialysis: first group (n = 23) included patients within their first 6 months of hemodialysis; group two (n = 55) with dialysis period between 6 months and 3 years and third group (n = 22) included those with more than three years period of hemodialysis.

Blood pressure before dialysis and interdialytic weight gain also recorded. Cardiovascular risk factors and demographic findings were noted. Three groups were matched for gender and cardiovascular risk factors. Hypertension was accepted when pre-dialysis blood pressures reached SBP ≥ 140 mmHg and/or DBP ≥ 90 mmHg.

### Laboratory data

Blood samples were collected pre-dialysis. Fasting serum samples were obtained in the early morning for biochemical studies. Most laboratory values including hemoglobin levels and serum levels of urea nitrogen (BUN), creatinine, electrolytes, calcium, phosphorus, total protein, albumin, total cholesterol, and triglycerides were measured by standard enzymatic procedures.

### Echocardiographic findings

Echocardiography was performed using M mode and two-dimensional ultrasonography. The protocol recommended that echocardiography be performed with the patient at dry weight, within 24 h after dialysis therapy in hemodialysis patients. All echocardiographic measurements were carried out according to the recommendations of the American Society of Echocardiography [13]. Echocardiographic findings including LVH, left atrial dilatation, mitral and aorta insufficiency and calcification and pleural effusion were compared between groups. The echocardiographic readings were performed on all patients by the same echocardiographist.

### Statistical analysis

Continuous data with normal distribution are given as mean ± standard deviation, otherwise as median, one-Way ANOVA test for testing the significance of mean for independent continuous scale data, Chi-square or Fisher exact test for testing the significance of percentages. A P value < 0.05 was considered significant. Statistical analyses were performed using the Statistical Package for Social Sciences, version 13.0 (SPSS, Chicago, Illinois).

## Results

Clinical data of the three patient groups are shown in Table 1. The three groups were comparable for gender, prevalence of diabetes, hypertension, hyperlipidemia and smoking, cardiovascular disease familial history, and hemodialysis period; the groups differed regarding age, which was significantly higher in third group (P < 0.005). Diastolic blood pressure as well as interdialytic weight gain was significantly higher in third group than two other groups (both P = 0.01).

**Table 1:** Clinical Findings Between Three Groups

	Group 1 (≤ 6 mnt)	Group 2 (>6 mnt ≤ 3 yrs)	Group 3 (> 3 yrs)	P value
Age (mean ± SD)	45.73 ± 13.25	52.50 ± 12.55	57.86 ± 9.44	0.005^*^
Gender (male%)	12 (52.2%)	25 (45.5%)	14 (63.6%)	0.35
Diabetics (No.) (%)	4 (17.4%)	9 (16.4%)	6 (27.3%)	0.53
Hypertensive (No.) (%)	3 (13%)	12 (21.8%)	7 (31.8%)	0.31
Hyperlipidemia (No.) (%)	7 (30.4%)	12 (21.8%)	10 (15.5%)	0.11
Current smoking (No.) (%)	2 (8.7%)	3 (5.5%)	2 (9.1%)	-----
Positive family history of CAD (No.) (%)	4 (17.4%)	6 (10.9%)	2 (9.1%)	0.64
Hemodialysis time (per week)	3.04 ± 0.20	2.78 ± 0.41	2.63 ± 0.49	0.003^*^
Systolic Blood pressure (mean ± SD)	131.51 ± 9.46	128.73 ± 9.72	133.64 ± 16.56	0.21
Diastolic Blood pressure (mean ± SD)	84.78 ± 9.22	80.72 ± 8.73	87.72 ± 12.22	0.01^*^
Weight gain (mean ± SD)	1.24 ± 0.56	1.09 ± 0.23	1.46 ± 0.82	0.01^*^

* P is two tailed significant.

FBS, creatinine, sodium, calcium, phosphorus, cholesterol and hemoglobin levels were comparable between three groups (Table 2). BUN and albumin levels significantly were higher and potassium levels were lower in first group (P = 0.01 and P = 0.007, P = 0.002 respectively).

**Table 2:** Laboratory Findings Between Three Groups

	Group 1 (≤ 6 mnt)	Group 2 (> 6 mnt ≤ 3 yrs)	Group 3 (> 3 yrs)	P value
Fasting blood sugar (mean ± SD)	119 ± 20.02	109.07 ± 22.35	118 ± 29.21	0.14
Blood urea nitrogen (mean ± SD)	70.78 ± 16.72	56.21 ± 22.19	64.27 ± 19.63	0.01^*^
Creatinine (mean ± SD)	5.18 ± 1.62	5.03 ± 1.96	6.27 ± 2.45	0.05
Albumin (mean ± SD)	3.59 ± 1.21	2.90 ± 0.70	2.89 ± 0.69	0.007^*^
Sodium (mean ± SD)	145.13 ± 3.92	144.47 ± 3.91	144.52 ± 5.17	0.81
Potassium (mean ± SD)	4.90 ± 0.51	5.62 ± 0.82	5.40 ± 0.85	0.002^*^
Calcium (mean ± SD)	7.90 ± 0.74	7.91 ± 0.92	7.47 ± 1.34	0.2
Phosphorus (mean ± SD)	4.35 ± 1.13	4.57 ± 1.41	4.96 ± 1.35	0.32
Cholesterol (mean ± SD)	144.26 ± 31.56	162.29 ± 42.46	168.05 ± 26.71	0.07
Hemoglobin (mean ± SD)	12.83 ± 0.88	12.28 ± 1.68	11.80 ± 1.96	0.1

* P is two tailed significant.

Table 3 shows echocardiographic findings between groups. LVEF was reduced by increase in dialysis period; LVH and LA dilatation as well as mitral insufficiency was higher in patients with higher period of dialysis; however these differences was not significant (P = NS). Other parameters were comparable between groups. Mitral calcification, aortic insufficiency and aortic calcification prevalence was < 5% so the results were not compared between groups.

**Table 3:** Echocardiographic Findings Between Groups

	Group 1 (≤ 6 mnt)	Group 2 (> 6 mnt ≤ 3 yrs)	Group 3 (> 3 yrs)	All	P value
LVEF ≤ 50% (No.) (%)	5 (21.7%)	14 (25.5%)	9 (40.9%)	28%	0.29
LVH (No.) (%)	4 (17.4%)	15 (27.3%)	7 (31.8%)	26%	0.51
LA dilation (No.) (%)	4 (17.4%)	15 (27.3%)	7 (31.8%)	26%	0.51
Diastolic dysfunction (grade III, IV) (No.) (%)	1 (4.3%)	15 (27.3%)	6 (27.3%)	22%	0.06
Mitral insufficiency (No.) (%)	13 (56.5%)	36 (65.5%)	18 (81.8%)	67%	0.18
Mitral calcification (No.) (%)	0%	3 (5.5%)	0%	3%	----
Aortic Insufficiency (No.) (%)	0%	2 (3.6%)	1 (4.5%)	3%	----
Aortic calcification (No.) (%)	0%	1 (1.8%)	0%	1%	----
Pericardial effusion (No.) (%)	4 (17.4%)	24 (43.6%)	7 (31.8%)	35%	0.08

LVEF: Left ventricular ejection fraction; LVH: Left Ventricle Hypertrophy; LA: Left atrium.

**Figure 1:**
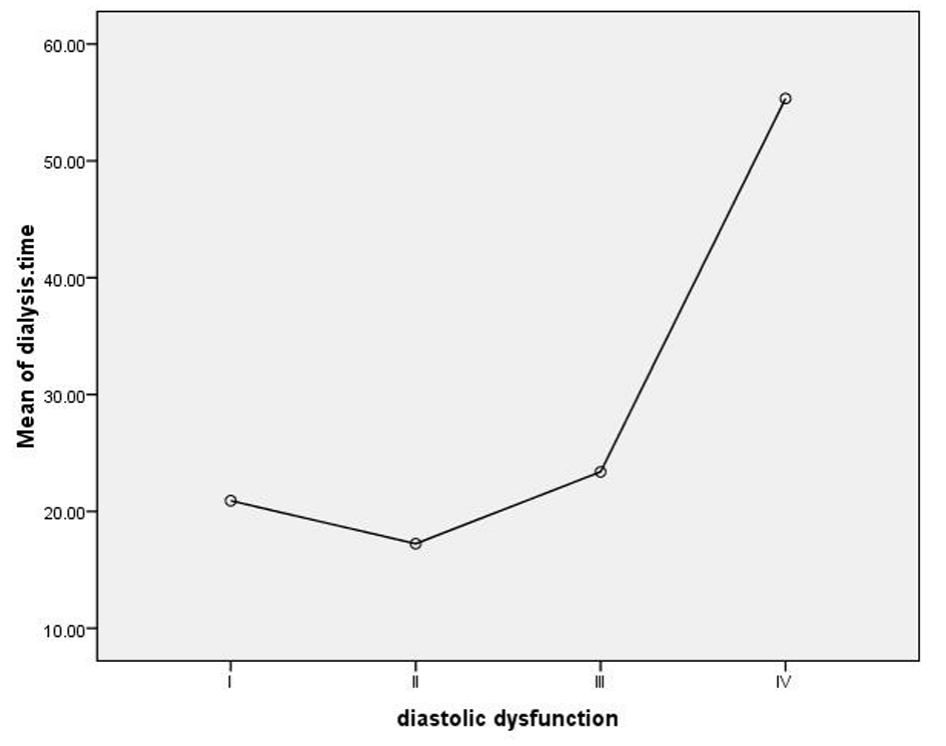
Mean hemodialysis period.

Mean dialysis period in patients with and without LVH was 34.80 ± 9.62 and 18.51 ± 2.22 months; Patients with LVH had significantly longer hemodialysis period (P = 0.01). Grade of diastolic dysfunction significantly increased by the dialysis period (P = 0.007), as in diastolic dysfunction grade IV mean hemodialysis period was more than 2 years (Fig. 1).

## Discussion

Although hemodialysis (HD) keeps alive patients with end-stage renal disease (ESRD), the survival of these patients is still reduced [14, 15], and despite technologic advances, it has not improved much over the last two decades [1, 16]. Cardiovascular diseases are the most important cause of mortality in HD patients, accounting for about 50% of deaths [1, 14], rendering the rate of cardiovascular mortality in these patients 20 times greater compared with that in the general population [17].

Left ventricular hypertrophy (LVH) and left ventricular systolic and diastolic dysfunction are the most common cardiovascular abnormalities and associated with increased morbidity and mortality in patients with ESRD [18]. In one study higher LVH, left ventricular diastolic dysfunction, valvular disease and calcification were reported in hemodialysis patients [19].

In this study we aim to compare time dependant echocardiographical findings in chronic hemodialysis patients in three different time periods. In our study of 100 hemodialysis patients, 26% had LVH and LA dilatation. Severe diastolic dysfunction was observed in 22%. Foley et al [7] studied the echocardiographic risk factors for the development of heart failure in patients who underwent echocardiography at baseline and at one year after starting dialysis therapy. In their study systolic dysfunction was observed in 15%, LA dilatation in 32% and LVH in 74% of patients; these were higher from our study.

In another study among patients with more than 3 months of hemodialysis, deFilippi et al [20] reported LVH in 46% of patients. In our study 2/3 of patients were under dialysis less than 3 years; a period that various treatments are used to prevent disease progression and severe complications. The difference between groups could be due to strategies to regulate blood pressure levels of the patients, treatment of anemia and other metabolic disorders during the HD period and the prevention of weight gain and hypervolemia, the risk factors related to LV function change [21, 22].

At a later stage longstanding haemodialysis is associated with advanced cardiac dysfunction [12, 23]. In patients with long time hemodialysis, LVH exist in three fourth of them and usually accompany by LA dilatation and LV dysfunction [7]. Also, heart chambers dilatation happens in long with compensated hypertrophy in time in dialysis patients [20].

Kocinaj and coworkers [24] reported that LA dilation was higher by increase in years of hemodialysis. Borsboom et al [25] reported high incidence of LVH and diastolic dysfunction mostly in patients being under dialysis for more than 3 years. However, in current study although not significant, LVH, LA dilation, reduced ejection fraction and mitral insufficiency prevalence were higher by increase in the time under hemodialysis. However, diastolic dysfunction significantly increased in time as severe diastolic dysfunction was higher in patients with more than 2 years of dialysis and patients with LVH had significantly longer hemodialysis period. Likewise, in the study of Duran and coworkers [26] LV systolic functions, LV diameters, LV mass index, left atrium size, and RV diastolic functions were not statistically different after long-term HD treatment.

### Conclusion

In hemodialysis patients, diastolic dysfunction increases by the hemodialysis time (years). LVH and LA dilation also increase during time, but not significantly. These findings could be due to a limitation; most patients were between 6 months to 3 years of hemodialysis which could affect our results.
